# Nitrate reduction capacity of the oral microbiota is impaired in periodontitis: potential implications for systemic nitric oxide availability

**DOI:** 10.1038/s41368-023-00266-9

**Published:** 2024-01-05

**Authors:** Bob T. Rosier, William Johnston, Miguel Carda-Diéguez, Annabel Simpson, Elena Cabello-Yeves, Krystyna Piela, Robert Reilly, Alejandro Artacho, Chris Easton, Mia Burleigh, Shauna Culshaw, Alex Mira

**Affiliations:** 1 Department of Genomics and Health, FISABIO Foundation, Center for Advanced Research in Public Health, Valencia, Spain; 2 Department of Biological and Biomedical Sciences, Glasgow Caledonian University, Glasgow, UK; 3 Oral Sciences, University of Glasgow Dental School, School of Medicine, Dentistry and Nursing, College of Medical, Veterinary and Life Sciences, University of Glasgow, Glasgow, UK; 4 Sport and Physical Activity Research Institute, University of the West of Scotland, Blantyre, Scotland; 5 Instituto de Biomedicina de Valencia, Consejo Superior de Investigaciones Científicas (IBV-CSIC), Valencia, Spain; 6 CIBER Center for Epidemiology and Public Health, Madrid, Spain

**Keywords:** Dental diseases, Microbiome, Physiology

## Abstract

The reduction of nitrate to nitrite by the oral microbiota has been proposed to be important for oral health and results in nitric oxide formation that can improve cardiometabolic conditions. Studies of bacterial composition in subgingival plaque suggest that nitrate-reducing bacteria are associated with periodontal health, but the impact of periodontitis on nitrate-reducing capacity (NRC) and, therefore, nitric oxide availability has not been evaluated. The current study aimed to evaluate how periodontitis affects the NRC of the oral microbiota. First, 16S rRNA sequencing data from five different countries were analyzed, revealing that nitrate-reducing bacteria were significantly lower in subgingival plaque of periodontitis patients compared with healthy individuals (*P* < 0.05 in all five datasets with *n* = 20–82 samples per dataset). Secondly, subgingival plaque, saliva, and plasma samples were obtained from 42 periodontitis patients before and after periodontal treatment. The oral NRC was determined in vitro by incubating saliva with 8 mmol/L nitrate (a concentration found in saliva after nitrate-rich vegetable intake) and compared with the NRC of 15 healthy individuals. Salivary NRC was found to be diminished in periodontal patients before treatment (*P* < 0.05) but recovered to healthy levels 90 days post-treatment. Additionally, the subgingival levels of nitrate-reducing bacteria increased after treatment and correlated negatively with periodontitis-associated bacteria (*P* < 0.01). No significant effect of periodontal treatment on the baseline saliva and plasma nitrate and nitrite levels was found, indicating that differences in the NRC may only be revealed after nitrate intake. Our results suggest that an impaired NRC in periodontitis could limit dietary nitrate-derived nitric oxide levels, and the effect on systemic health should be explored in future studies.

## Introduction

The accumulation of dental plaque due to insufficient oral hygiene can facilitate the development of gingivitis.^1^ Gingivitis is mostly reversible, but long or repeated episodes of gingivitis, especially in susceptible individuals, can lead to the development of periodontitis—a chronic and destructive inflammatory disease in which host tissue is lost. In periodontitis, periodontal pockets develop in which the subgingival plaque microbiota shifts towards a disease-associated composition, including an increase of anaerobic, proteolytic, inflammation-tolerant, and/or alkalophilic species.^2–4^

Along with an increase in disease-associated bacteria, a decrease in health-associated bacteria has also been observed.^5,6^ A healthy subgingival environment has normally been associated with the dominance of aerobic or facultatively anaerobic organisms.^3^ However, closer investigation of these healthy microbial populations reveals that they include all genera that to date have been confirmed to reduce nitrate by physiological measurements, namely *Rothia*, *Neisseria*, *Actinomyces*, *Veillonella*, *Kingella*, and *Propionibacterium*.^7^ Of these genera, *Rothia* and *Neisseria* are the bacteria with the strongest association with nitrate, increasing in most (if not all) studies in which oral communities are exposed to nitrate.^8,9^ It has been estimated that we obtain over 80% of dietary nitrate from vegetables—a food group strongly associated with systemic health benefits. However, the relationship between periodontitis, nitrate-rich foods, and systemic health consequences has yet to be elucidated.

For centuries, it has been known that the impact of periodontitis is not isolated to the oral cavity, where it can cause inflammation, bleeding, halitosis, and tooth loss. The oral cavity is the beginning of the respiratory system and gastrointestinal tract and is directly connected to the bloodstream via highly vascularized oral tissues.^10^ Periodontitis is associated with an increased risk of diabetes, rheumatoid arthritis, atherosclerosis, hypertension, pregnancy complications, and Alzheimer’s disease, among others.^11^ This periodontal-systemic link gave rise to the concept of periodontal medicine,^12^ which, from a mechanistic point of view, has been explained as the effect of periodontitis-associated bacteria, their products, and/or inflammatory molecules produced in the inflamed gingiva reaching different parts of the body and causing complications through a systemic inflammation state.^10,11^

In contrast, some oral microbiota products can result in health benefits. For example, nitrate-reducing bacteria in the mouth reduce nitrate to nitrite and, in some cases, further to nitric oxide.^13,14^ The nitrite and nitric oxide produced by oral bacteria can enter the bloodstream directly via the oral mucosa.^15^ Equivalent to the mode of action of nitroglycerin (a nitric oxide liberator) tablets, which are placed below the tongue for direct entrance to the bloodstream, nitrite and nitric oxide produced by subgingival bacteria can enter the circulation via the gingival crevice. Indeed, in a recent study by Goh et al., the nitrite production of subgingival plaque was associated with cardiovascular health, indicating that this community could contribute to systemic nitric oxide levels.^16^ Additionally, nitrite produced by the oral microbiota is swallowed and converted into nitric oxide in the stomach (by acidic decomposition of nitrite) and host tissue (e.g., by the reaction of nitrite with hemin).^17^ This so-called nitrate-nitrite-nitric oxide pathway is oral microbiota dependent and can have several cardio-metabolic benefits when stimulated with dietary nitrate, including lowering of blood pressure, improved endothelial function, reversal of metabolic syndrome, antidiabetic effects, and enhanced exercise performance under certain circumstances.^18–20^ Therefore, it is crucial to evaluate if, in addition to the pro-inflammatory effect of periodontal pathogens, a lowered capacity to utilize dietary nitrate could be taking place during periodontitis which could also contribute to the systemic effects of this disease.

In addition to systemic benefits, nitrate metabolism by the oral microbiota appears to be important for oral health (reviewed by Rosier et al.^7^). Nitric oxide has antimicrobial properties, killing sensitive species, including anaerobic bacteria associated with periodontitis.^21^ In contrast, representatives of *Rothia* and *Neisseria* increase in the presence of nitrate and are associated with the absence of inflammation.^7^ Nitric oxide could also directly signal to epithelial cells, possibly contributing to gingival homeostasis by increasing blood flow and mucus thickness while decreasing inflammation.^17,22,23^

In the current work, we aim to establish if the nitrate-nitrite-nitric oxide pathway could be impaired in periodontitis. Additionally, we aim to determine the effect of periodontitis and periodontal treatment on the nitrate-reducing subgingival microbiota. For this, 16S rRNA sequencing data of subgingival samples were analyzed to determine if nitrate-reducing bacteria were lower in periodontitis compared to periodontal health. Additionally, subgingival plaque, saliva and plasma samples were collected from periodontitis patients before and 90 days after periodontal treatment. Nitrate and nitrite were measured in saliva and plasma, and the salivary nitrate reduction capacity (NRC) was determined by incubating saliva with a physiological nitrate concentration. To examine the effect of periodontal treatment on nitrate-reducing bacteria in subgingival plaque, the levels of nitrate-reducing species were determined using Illumina sequencing data and the genus *Rothia*, which can be considered a biomarker of nitrate reduction,^7,24^ was measured by qPCR.

## Materials and methods

### Bioinformatic analysis comparing health and periodontitis

**Fig. 1:**
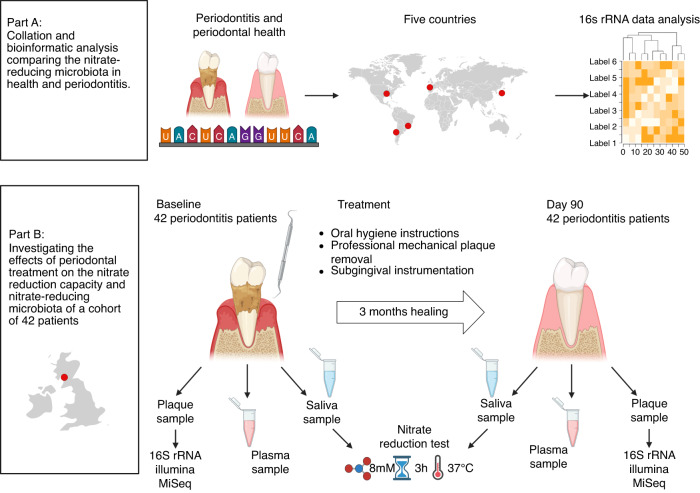
Study overview. Part A: Bioinformatic analysis comparing health and periodontitis. 16S rRNA sequencing data of five studies from five different countries (Japan,^25^ Brazil,^26^ Chile,^27^ USA^28^ and Spain,^29^ *n* = 20–82 samples per study) were analyzed with the Dada2 pipeline to compare nitrate-reducing bacteria in health and periodontitis. Part B) Sample analysis of periodontitis treatment study. Subgingival plaque, saliva, and plasma samples from 42 periodontitis patients located in Glasgow (Scotland, United Kingdom) were collected before and 90 days after periodontal treatment. The subgingival plaque bacterial composition was determined using Illumina sequencing of the 16S rRNA gene, and the quantity of the nitrate-reducing biomarker genus *Rothia* was determined by qPCR. Measurements of nitrate and nitrite in saliva and plasma were performed and the salivary nitrate reduction capacity (NRC) was determined after three hours of incubation in vitro and compared with the NRC of 15 healthy individuals

To evaluate if the total level of nitrate-reducing bacteria could decrease in periodontitis, bioinformatic analysis of five datasets was performed to find differences in the nitrate-reducing microbiota between periodontal health and periodontitis (Fig. 1A). Specifically, studies were selected where the average probing depth (entire mouth or sampled sites) of the periodontitis individuals was at least two times higher than the healthy group (Supplementary Table 1). Datasets containing 16S rRNA sequencing data from subgingival plaque samples were downloaded from the NCBI SRA Database and included individuals from different countries (Japan,^25^ Brazil,^26^ Chile,^27^ USA^28^, and Spain^29^), taking into account that the country of origin can have a significant effect on microbiota composition.^30^ The number of individuals and samples, as well as the criteria to describe health and periodontitis in these studies, are presented in Supplementary Table 1. The FastQ files were processed as previously described using the DADA2 R Statistics package (v1.20.0).^31,32^ In summary, R1 and R2 reads were trimmed by length, and reads with more than 5 errors were removed. Reads were dereplicated to obtain true sequence variants that were then merged (min. overlap 15 bp) and annotated to the SILVA v.138.1 database.^33,34^ Bacteria were classified as nitrite-producers or confirmed nitrate reducers based on Rosier et al.^7^ Additionally, species were classified as periodontitis-associated, including the red and orange complexes identified by Socransky et al.^35^ and periodontitis-associated bacteria identified by Perez-Chaparo et al.^36^ or just red complex. The exact list of species in the four groups (nitrite producers, confirmed nitrate reducers, periodontitis-associated, and red complex) can be found in Supplementary Table 2.

### Bacterial composition in periodontitis patients before and after periodontal treatment

To study the effect of periodontal treatment on the nitrate-reducing microbiota, data and samples were used from a study previously described by Davison et al.^37^ and Johnston et al.^38^ The study was conducted in accordance with the Declaration of Helsinki (2013) and received ethical approval (London-Stanmore Research Ethics Committee, Reference: 14/LO/2064). The patients were recruited at the Glasgow Dental Hospital and periodontitis was defined as probing pocket depths ≥ 5 mm on 2 or more teeth at non-adjacent sites excluding third molars. All patients signed a written informed consent prior to participation. Other inclusion criteria and sample collection are described in Johnston et al.^38^ In short, 42 patients were included and received periodontal treatment, including professional mechanical plaque removal (PMPR) and subgingival instrumentation^39^ at all sites requiring this (previously referred to as non-surgical periodontal treatment, NSPT^37,38^), by a single experienced dental hygienist in one to six visits. Post hoc analysis of these individuals using recent periodontitis guidelines,^40^ revealed that the majority were diagnosed with generalized periodontitis (*n* = 40), mostly of stage II or III (*n* = 38).^41^ Subgingival plaque, drooling saliva, and plasma samples were taken before any treatment visits (baseline, BL) and 90 days following the last treatment visit (day 90, D90). A period of 90 days was selected based on the time needed for healing and reestablishment of subgingival communities,^42^ supported by current treatment guidelines.^39,43^ At day 90, periodontal parameters had improved significantly (e.g., the periodontal inflamed surface area, PISA, decreased significantly from a median of 1225 mm^2^ at BL to 139 mm^2^ at D90).^38^ Additionally, healthy control saliva samples were collected from volunteers under the study “Host-microbiota interactions in oral health and disease”, project number 2011002. The healthy control study received ethical approval from the University of Glasgow MVLS ethical committee. All samples were stored at −80 °C prior to usage.

The DNA extraction and Illumina sequencing were performed at the FISABIO Foundation (Valencia, Spain) as previously described by Johnston et al.^38^ DNA was extracted from subgingival samples (baseline and day 90) using the MagNA Pure LC DNA isolation kit (Roche Diagnostics, Mannheim, Germany) with the addition of a chemical lysis step with an enzymatic cocktail containing lysozyme, mutanolysin and lysostaphin, following Rosier et al.^9^ As previously described by Johnston et al.^38^ DNA concentrations were measured using a QubitTM 3 Fluorometer (Thermofisher, Waltham, Massachusetts, USA). An Illumina amplicon library was prepared following the 16S rRNA gene Metagenomic Sequencing library preparation Illumina protocol (Part #15,044,223 Rev. A). The primer sequences used in this protocol were; Illumina_16S_341F (TCGTCGGCAGCGTCAGATGTGTATAAGAGACAGCCTACGGGNGGCWGCAG) and Illumina_16S_805R (GTCTCGTGGGCTCGGAGATGTGTATAAGAGACAGGACTACHVGGGTATCTAATCC) which target the 16S V3 and V4 region. Following amplification, DNA was sequenced with an Illumina MiSeq Sequencer according to the manufacturer’s instructions using the 2 × 300 base paired-ends protocol. For taxonomic classification, an amplicon sequence variant (ASV) table was obtained using the DADA2 pipeline in R.^31^ Taxonomy was assigned by comparison to the SILVA database,^33^ where the naive Bayesian classifier was used to assign sequences at the species level. In the current study, a new analysis is presented in which bacterial species were classified as confirmed nitrate-reducers or nitrite-producers (Supplementary Table 2).

### qPCR of Rothia in subgingival plaque

The subgingival plaque DNA was used for quantitative PCR (qPCR) measurements to determine the total amount of *Rothia* cells. Specifically, the *Rothia* nitrate reductase *narG* gene was amplified as described by Rosier et al.^24^ Primers sequences were designed to be specific for the *Rothia* genus, using conserved regions of *narG* from *Rothia mucilaginosa*, *R. dentocariosa,* and *R. aeria*. The forward primer sequence was 5’-ACA CCA TYA AGT ACT ACGG-3’ and the reverse 5’-TAC CAG TCG TAG AAG CTG-3’. Reactions of 20 μL were added per well of a qPCR plate, consisting of 10 μL of Light Cycler 480 SYBR Green I Master mix (Roche Life Science, Penzberg, Germany), 0.4 μL of each specific primer (10 μmol/L), 8.2 μL water and 1 μL of template DNA (DNA isolated from subgingival plaque samples). Each sample was added in duplicate, and measurements were performed using a Light Cycler 480 Real-Time PCR System (Roche Life Science) with the following conditions: 95 °C for 2 min, 40 cycles of 95 °C for 30 s, 60 °C for 20 s, and 72 °C for 25 s. Negative controls were added, as well as a standard curve, consisting of a series dilution of an equimolar DNA mix of three *Rothia* species (*R. mucilaginosa* DSM-20746, *R. dentocariosa* DSM- 43762, *R. aeria* DSM-14556) quantified with a QubitTM 3 Fluorometer (Thermofisher). Based on genome sizes,^44^ the number of *Rothia* cells was calculated, assuming a single copy of the *narG* gene per cell. The samples of five individuals out of 42 individuals had no DNA left after the sequencing procedure, leading to qPCR data of 37 individuals.

### Salivary nitrate reduction test

The NRC in periodontal patients before and after periodontal treatment (*n* = 42) and healthy controls (*n* = 15) was determined by defrosting saliva samples on ice and incubating them for 3 h at 37 °C in the presence of 8 mmol/L nitrate. For this, 25 μL of water with 80 mmol/L sodium nitrate (Sigma) was added to 225 μL of saliva in an Eppendorf tube.

### Nitrate, nitrite, and pH measurements in saliva

For nitrate, nitrite and pH measurements in saliva, the RQflex 10 Reflectoquant (Merck Millipore, Burlington, Massachusetts, USA) reflectometer was used as described by Rosier et al.^9^ The test strips (Reflectoquant, Merck Millipore) for nitrate had a range of 3–90 mg/L, the strips for nitrite a range of 0.5–25 mg/L, and the strips for pH a range from pH 4–9. The accuracy of these reflectometer methods was confirmed using standard solutions (Merck Millipore) with known concentrations of nitrate and nitrite or pH levels. For pH measurements, undiluted saliva was used. For nitrate and nitrite measurements, the saliva was used directly or diluted 5-10 fold depending on the compound concentrations. Fifteen microlitres of (diluted) saliva were added to each of the two reactive patches on a strip, and excess liquid was removed by tipping the side of the strip on a tissue.

Before nitrate measurements, the diluted supernatants were treated with amidosulfuric acid (Sigma-Aldrich) based on the manufacturer’s instructions. For this, 35 µL of diluted supernatant was mixed with 1.5 µL amidosulfuric acid solution (10%). The nitrate, nitrite, and pH measurements were performed before in vitro incubation (basal levels) and after 3 hours of incubation to determine the NRC (post-incubation levels).

### Nitrate and nitrite measurements in plasma

The basal plasma nitrate and nitrite levels were determined before and after periodontal treatment using ozone-based chemiluminescence as described by Liddle et al.^45^ For the measurement of plasma nitrite, tri-iodide reagent and 100 μL of anti-foaming agent were placed into a purge vessel which was heated to 50 °C. A standard curve was produced by injecting 100 μL of nitrite solutions (62.5–1 000 nmol/L) and a control sample (0 nmol/L). Following this, plasma samples were thawed in a water bath at 37 °C for 3 min, and 100 μL of the sample was injected into the purge vessel in duplicate. The concentration of NO cleaved during the reaction was then measured by the NO analyzer (Sievers NOA 280i, Analytix, UK), for the measurement of plasma nitrate, vanadium reagent, and 100 μL of the anti-foaming agent were placed into the glass purge vessel, and heated to 95 °C. A standard curve was produced by injecting 25–50 μL of nitrate solutions (6.25–100 μmol/L) and a control sample (0 μmol/L). Plasma samples were thawed and de-proteinised. Subsequently, 50 μL of the sample was injected into the purge vessel in duplicate, and plasma nitrate was calculated as previously described for the nitrite assay.

### Statistical analysis

Statistical analysis of nitrate and nitrite in saliva and plasma and *Rothia* cells in subgingival plaque (determined by qPCR) was performed using a nonparametric Wilcoxon test using IBM SPSS statistics (version 27) or GraphPad (version 9.5.1) and considered statistically significant at *P*-value < 0.05.

For analysis of the bacterial groups (i.e., nitrite producers, confirmed nitrate reducers, periodontitis-associated, and red complex), R programming language (v3.4+) was used for statistical computing. The abundance of species was standardized using ANCOM-BC.^46^ The sums of standardized compositional data of bacteria in different groups were then compared using the Wilcoxon signed rank tests (Wilcox.test function of stats library of R) and considered statistically significant at adjusted *P*-value < 0.05 (i.e., corrected for multiple comparisons using the Benjamini–Hochberg false discovery rate (FDR) of 5%).

Correlations within and between the relative abundances of groups of bacteria and physiological parameters were determined with Spearman’s rho (cor.test function of stats library of R), along with associated adjusted *P*-values. Apart from the parameters of this study, correlations with salivary cytokines [tumor necrosis factor α (TNFα), interleukin-6 (IL-6) and interleukin-1β (IL-1β)] and clinical parameters [Periodontal pocket depths (PPD), clinical attachment level (CAL), full mouth bleeding score (FMBS), full mouth plaque score (FMPS) and PISA] obtained by Johnston et al.^38^ were explored.

**Fig. 2:**
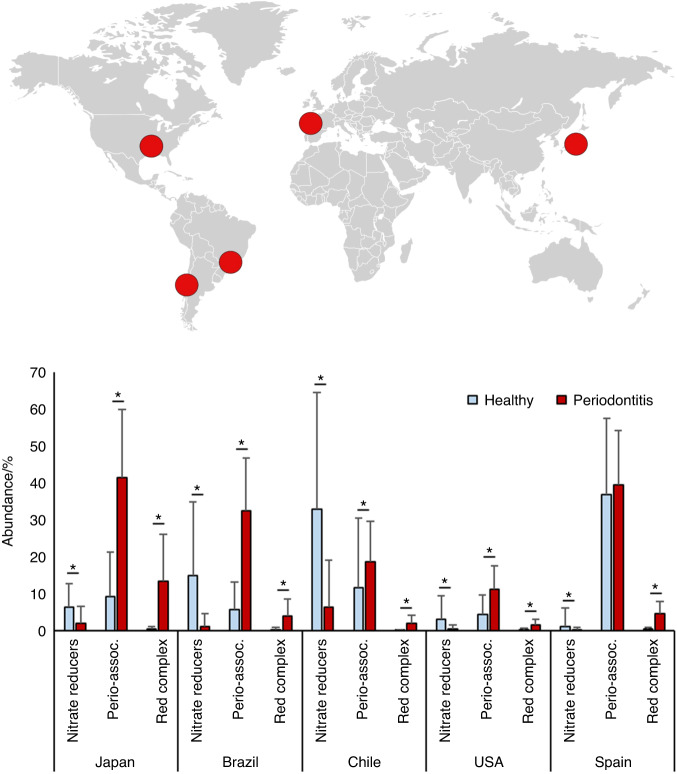
Confirmed nitrate-reducing bacteria in periodontitis and health. Bar graphs show the relative abundance of bacteria in subgingival plaque samples from different countries, as estimated by high-throughput sequencing of the 16 S rRNA gene. Bacteria were grouped in confirmed nitrate-reducing species, periodontitis-associated or red complex according to Rosier et al.^7^, Pérez-Chaparro et al.^36^, and Socransky et al.^35^ respectively (the bacterial species in each group are listed in Supplementary Table 2). Healthy individuals (blue bars) were compared with individuals with periodontitis (red bars). In Supplementary Fig. 1, all known nitrite-producing bacteria (some of which may produce nitrite by pathways other than nitrate reduction) are shown following the same pattern and showing a significant difference between health and periodontitis. The datasets include individuals from Japan, Spain, the USA, Brazil, and Chile (*n* = 20–82 per dataset, see Supplementary Table 1 for additional information). It should be noted that the levels of groups of bacteria in different studies are affected by the criteria used to describe periodontitis, regional and host factors, as well as the DNA extraction methods or the sequencing techniques used in the original studies. *adjusted *P* < 0.05 of compositional data standardized by ANCOM-BC and compared with a Wilcoxon test

Figures 1 and 2 and Supplementary Fig. 1 were created in Microsoft Excel and/or using BioRender, and all other Figures were assembled using GraphPad PRISM (version 9.5.1).

## Results

### Levels of nitrate-reducing bacteria in periodontal patients

A total of datasets of five different countries were analyzed, where 16S rRNA gene sequencing of subgingival samples was performed (clinical information of the individuals in each dataset is found in Supplementary Table 1). These datasets spanned individuals in Japan (*n* = 10 periodontitis samples and 10 healthy samples), Spain (*n* = 60 periodontitis samples and 22 healthy samples), USA (n = 29 periodontitis samples and 29 healthy samples), Brazil (*n* = 27 periodontitis samples and 21 healthy samples), and Chile (*n* = 22 periodontitis samples and 17 healthy samples). As expected, a higher proportion of red complex bacteria directly involved in periodontitis was found in periodontal patients compared to healthy individuals in all five countries (Fig. 2). In relation to the denitrifying microbiota, the relative proportion of nitrate-reducing bacteria varied between datasets. Regardless of the levels in healthy individuals in each country, the proportion of nitrate-reducing bacteria was significantly lower in periodontal patients in all cases (*P* < 0.05 in all countries) (Fig. 2). When considering all known nitrite-producing bacteria (some of which may produce nitrite by other pathways than nitrate reduction), the pattern and statistical significance remained (Supplementary Fig. 1).

### Levels of nitrate-reducing bacteria before and after periodontal treatment

**Fig. 3:**
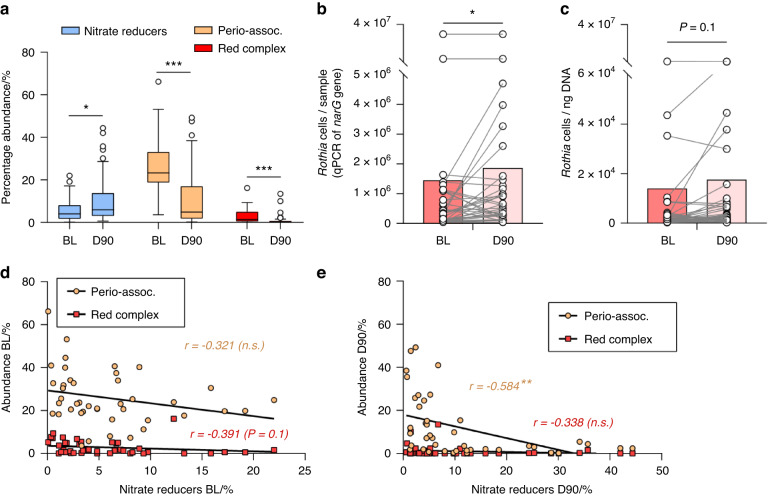
Confirmed nitrate-reducing species and disease-associated bacteria before and 90 days after periodontal treatment. **a** Relative abundances of confirmed nitrate-reducing bacteria, red complex, and periodontitis-associated bacteria before (baseline, BL) and 90 days after treatment (D90) of 42 periodontitis patients. *adjusted *P* < 0.05, ***P < 0.001 of compositional data standardized by ANCOM-BC and compared with a Wilcoxon test. **b**, **c** *Rothia* cells determined by qPCR before (BL) and 90 days after (D90) treatment per sample (absolute amount, *n* = 37) (**b**) or normalized per ng of DNA (**c**). *P < 0.05 determined by a Wilcoxon test. **d**, **e** Correlations between the abundance of periodontal pathogens and nitrate-reducing bacteria at baseline (BL) and 90 days after treatment (D90). **adjusted *P* < 0.01 of Spearman’s rank correlation (n.s. = not significant). In Supplementary Fig. 2, the comparisons and correlations of all known nitrite-producing bacteria (some of which may produce nitrite by other pathways than nitrate reduction) are shown

The bacterial composition of subgingival samples before (BL) and after (D90) periodontal treatment showed a consistent pattern across individuals. Relative to baseline, there was a significant increase in bacteria capable of producing nitrite (Supplementary Fig. 2), including confirmed nitrate-reducing bacteria (Fig. 3A), with an opposite pattern for periodontal pathogens, including both the three species of the red complex and the extended list of periodontitis-associated bacteria, as reported previously by Johnston et al.^38^ This suggests that the capacity to metabolize nitrate by subgingival plaque is improved after periodontal treatment. The increase in the levels of nitrate-reducing bacteria was confirmed by qPCR using specific primers for the nitrate-reducing biomarker *Rothia*, which showed a significant increase in cells per sample (i.e., absolute levels) (Fig. 3B). The normalized levels of this organism (*Rothia* cells determined by qPCR per ng DNA) also appeared to increase, but this increase was not significant (*P* = 0.1) (Fig. 3C). Notably, it was previously shown that the relative abundance of *Rothia* determined by Illumina sequencing did increase after periodontal treatment.^38^ Both the absolute and normalized levels of *Rothia* determined with qPCR correlated with the abundance of nitrate-reducing bacteria (*r* = 0.592 and *r* = 0.574, respectively, both *P* < 0.001 when grouping BL and D90) (Supplementary Datasheet).

When grouping the BL and D90 samples, a significant negative correlation was found between the levels of nitrate-reducing bacteria and the levels of periodontitis-associated bacteria (*r* = −0.523, *P* < 0.001) (Supplementary Datasheet). When considering the BL samples only, this correlation was not significant (Fig. 3D), but in the D90 samples, there was a clear negative correlation between nitrate-reducing bacteria and periodontitis-associated bacteria (*r* = −0.584, *P* < 0.01) (Fig. 3E). Two other interesting negative correlations when grouping the BL and D90 samples were between nitrate-reducing bacteria and FMPS (*r* = −0.383, *P* < 0.01), as well as absolute or total *Rothia* levels and FMBS (both *r* = −0.32 to 0.33, both *P* < 0.05). Finally, the group of all nitrite-producing bacteria (Supplementary Table 2) correlated negatively with salivary IL-1β at baseline (*r* = −0.547, *P* < 0.01) (Supplementary Datasheet).

### NRC in health and periodontitis

**Fig. 4:**
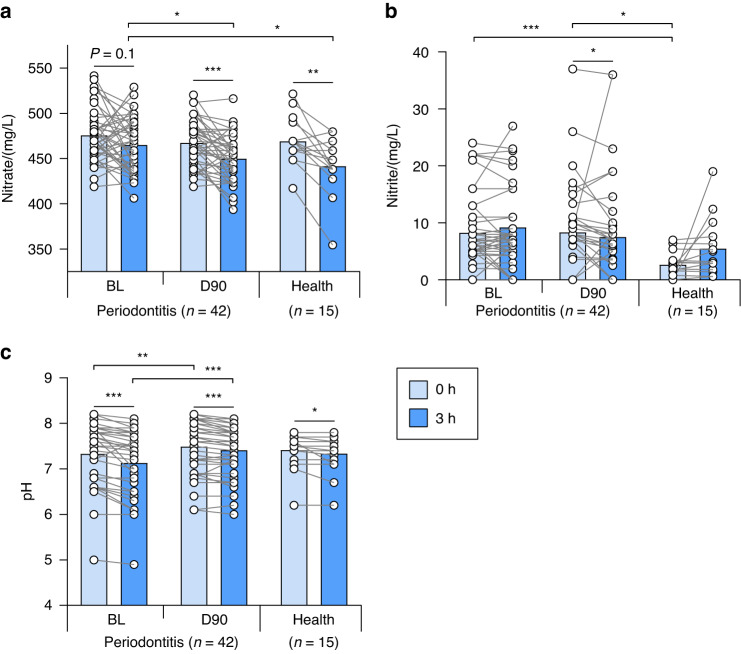
Nitrate reduction capacity (NRC) in health and periodontitis. **a**, **b** Bar graphs represent the concentration of nitrate (NO_3_^−^) and nitrite (NO_2_^−^) of saliva samples cultured in vitro for 3 h in the presence of 8 mmol/L nitrate at 37 °C before (Baseline, BL) and 90 days after periodontal treatment (*n* = 42) of periodontal patients, compared to healthy controls (*n* = 15). **c** Bars show the pH during this incubation period for the same groups of individuals. Asterisks represent statistically significant differences (*P < 0.05, **P < 0.01, ***P < 0.001) determined by a Wilcoxon test (paired) or a Mann–Whitney *U*-test (unpaired)

Nitrate utilization during the incubation of saliva samples with added nitrate for a period of three hours was considered an estimate of the NRC in each individual. The data revealed that nitrate reduction was higher in healthy individuals compared to periodontal patients at baseline (Fig. 4A): in periodontitis patients, nitrate levels did not change significantly over the 3-h incubation period (*P* = 0.1), while in healthy individuals, it did (*P* < 0.01). In accordance with the 16S rRNA sequencing data that showed that nitrate-reducing bacteria increased in subgingival plaque, the NRC in periodontal patients was restored after the periodontal treatment, with a significant depletion in nitrate levels after the incubation (*P* < 0.001).

The nitrate-reducing activity (a known pH buffering process) was also confirmed indirectly by pH measurements, which showed a slightly larger pH drop in periodontal patients at baseline than after treatment (at BL the pH dropped from 7.33 to 7.13, and at D90, it dropped from 7.49 to 7.41, both *P* < 0.001) (Fig. 4C).

In agreement with the above, nitrite levels did not differ in baseline periodontal samples before and after the incubation with nitrate (Fig. 4B). After periodontal treatment, a significant decrease in nitrite was observed after the incubation. Given the nitrate consumption during this three-hour period, the lower nitrite levels were probably a consequence of further nitrite metabolization. In healthy individuals, nitrite levels did increase after the incubation, but this increase was not significant (*P* = 0.17) and a much lower initial concentration of nitrite was present compared with periodontitis before (*P* < 0.001) and after (*P* < 0.05) treatment.

### Plasma levels of nitrate and nitrite

**Fig. 5:**
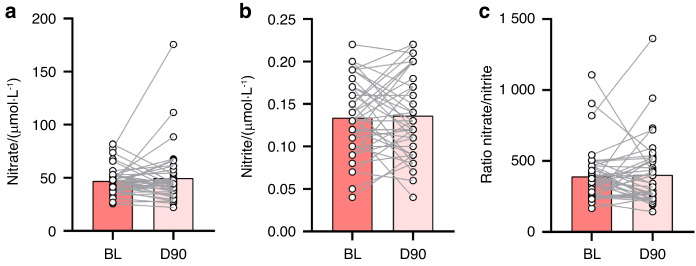
Plasma nitrate and nitrite before and 90 days after periodontal treatment. Data is shown from 42 periodontitis patients before (BL) and after (D90) treatment. **a** Plasma nitrite. **b** Plasma nitrate. **c** Ratio nitrate/nitrite

In agreement with the basal salivary levels of nitrate and nitrite, the basal nitrate and nitrite concentrations in blood samples collected in periodontal patients at baseline and 90 days after the periodontal treatment revealed no statistical differences between these two-time points (Fig. 5A, B). The average plasma nitrate levels were 47 μmol/L at BL and 49.7 μmol/L at D90 (not significantly different). Nitrite levels (134 nmol/L at BL and 136 nmol/L at D90) were around 350 times lower than nitrate (Fig. 5C). There were no significant correlations between bacterial groups and plasma nitrate or nitrite. The highest Spearman correlation was between absolute or normalized *Rothia* levels in subgingival plaque and plasma nitrite, but this correlation was not significant (both *r* = 0.278, both *P* = 0.1 when grouping BL and D90) (Supplementary Datasheet). The increased NRC that was found in saliva with added nitrate would translate into higher plasma nitrite or nitric oxide levels after dietary intake of nitrate, which was not evaluated in this study.

## Discussion

The nitrate-nitrite-nitric oxide pathway can result in cardiovascular and metabolic benefits when stimulated with dietary nitrate. In this study, we show that the reduction of 8 mmol/L nitrate (i.e., a concentration found in saliva after vegetable intake) by microorganisms in saliva was impaired in periodontitis. This impaired NRC was recovered to healthy levels after periodontal treatment. In line with this, we show that the levels of nitrate-reducing bacteria in subgingival plaque are lower in periodontitis and recovered after periodontal treatment. Our results indicate that an impaired NRC in periodontitis could limit the positive effects of dietary nitrate-derived nitric oxide. Notably, different systemic conditions associated with periodontitis are also associated with a deficit in nitric oxide (e.g., cardiovascular diseases and diabetes). Therefore, future studies should explore the effect of periodontitis and periodontal treatment on cardiometabolic parameters after dietary nitrate intake.

### The effect of periodontitis and periodontal treatment on the nitrate-reducing oral microbiota

To compare the subgingival levels of nitrate-reducing bacteria in health and periodontitis, we used 16S sequencing datasets of five different countries (Japan, Brazil, Chile, USA, and Spain). All countries followed the same pattern of a decrease in periodontitis-associated species and an increase in nitrite-producing bacteria, including confirmed nitrate-reducing species. This finding is in agreement with previous studies. For example, Feres et al.^5^ systemically reviewed findings from sequencing studies comparing subgingival plaque in health and periodontitis, as well as periodontitis before and after treatment and found that common nitrite-producing species were associated with periodontal health (e.g., *Steptococcus* spp., *Neisseria longate*, *Neisseria subflava*, *Rothia aeria*, *Veilonella Parvula* and *Granulicatella adiacens*). Additionally, Feres et al. found that *Rothia* was the genus with the strongest association with periodontal health, and with reduced inflammation after periodontal treatment, followed by other nitrite-producing genera (e.g., *Neisseria*, *Actinomyces* and *Steptococcus*). These results are further supported by bioinformatic analyses of Meuric et al.^47^ associating *Veillonella*, *Neisseria*, *Rothia*, *Corynebacterium*, and *Actinomyces* (all genera with nitrite-producing representatives) with periodontal health, and Chen et al.^6^ who reported that that *Steptococus sanguinis*, *Actinomyces naeslundii*, *Rothia aeria*, *Granulicatella adiacens*, *Rothia dentocariosa* and *Streptococcus mitis* (all nitrite-producing species) were six of the top seven most health-associated species that are used to calculate the subgingival microbial dysbiosis index (SMDI).

The underlying mechanisms by which nitrate reduction by oral bacteria appears to be beneficial for oral health requires exploration.^7^ Oral bacteria can further reduce nitrite to nitric oxide, a free radical with antimicrobial properties capable of inhibiting sensitive species such as anaerobes associated with periodontitis.^21^ Additionally, nitric oxide derived from denitrification by oral bacteria could directly signal to human cells, reducing inflammation and stimulating protective mucus production and blood flow.^17,22,23^ In our study, nitrate-reducing bacteria correlated negatively with periodontitis-associated bacteria, supporting the idea that this function could be important for oral health. This negative correlation was clearest in the 42 periodontitis patients after periodontal treatment. The reduction of inflammation in these patients after treatment was previously described by Johnston et al.^38^ and consisted of a clear and significant improvement of all periodontal clinical parameters. In our recent in vitro study, the addition of nitrate to periodontal plaque resulted in nitrite production (an indication of nitric oxide production), a decrease in periodontitis-associated species, and a lower SMDI.^48^ In our current study, the levels of nitrate-reducing bacteria in subgingival plaque correlated negatively with the full-mouth plaque score (FMPS), while nitric oxide is also a biofilm dispersal signal for some bacteria that could reduce plaque accumulation.^2^ In summary, the impairment of the NRC in periodontitis could give a selective advantage to periodontitis-associated species by losing nitric oxide production and potentially other antimicrobial mechanisms of health-associated bacteria. The effect of nitrate consumption and the patients’ NRC on the efficiency of periodontal treatment should be further investigated in future studies.

One of the main results of this study is that the NRC of saliva samples decreases in periodontitis. Unstimulated saliva contains 10^8^–10^9^ bacteria per mL that have dispersed from oral communities,^49^ making this a non-invasive sample that can be used to study the activity of the oral microbiota. Additionally, by freezing at −80 °C and thawing on ice, a significant part of the bacteria survives, as demonstrated by the changes in metabolic parameters after 3 h of incubation at 37 °C in this study (e.g., a minor but significant decrease in pH by bacterial fermentation of salivary components). By adding 8 mmol/L nitrate to these saliva samples, significant nitrate-reduction activity was found in the saliva of 15 healthy individuals (*P* < 0.01), which is commensurate with previous data obtained in small cohorts of healthy populations (*n* = 9–12).^9,44^ Notably, no significant nitrate reduction was observed in the saliva of 42 periodontitis patients before treatment (*P* = 0.1). However, 90 days after treatment, the NRC of the individuals with periodontitis was restored (*P* < 0.001) to levels comparable with healthy individuals. Additionally, more nitrite was reduced after periodontal treatment, indicating that nitric oxide production could be increased. This could be explained by the observation that common nitrate-reducing bacteria, which increased in number, also have nitrite-reduction genes.^44^

The periodontal treatment had no significant effect on basal plasma nitrate and nitrite levels, while the effect of nitrate intake on plasma was not evaluated in this study. Specifically, the average basal plasma concentrations of nitrate in individuals with periodontitis were 47.0 μmol/L at BL and 49.7 μmol/L at D90 after treatment. The average nitrite levels were around 350 times lower (134 nmol/L at BL and 136 nmol/L at D90). In two studies with systemically healthy individuals recruited at the same location (Glasgow, Scotland, United Kingdom), similar plasma nitrate and nitrite levels were found in a fasting state: Burleigh et al.^50^ found averages of 34 μmol/L nitrate and 133 nmol/L nitrite in 25 individuals, while Liddle et al.^51^ found averages of 54 μmol/L nitrate and 137 nmol/L nitrite in 34 individuals. It is known that nitrate intake increases the plasma nitrate and nitrite concentrations multiple times,^17^ indicating that at the timing of sample collection in our study, no dietary nitrate metabolism was detected. Future studies in larger populations should determine if periodontitis could affect the levels of plasma nitrite and nitrate while fasting or after nitrate intake.

In summary, the basal levels of nitrate and nitrite in plasma and saliva were not affected by periodontal treatment, but a difference in NRC was revealed when 8 mmol/L nitrate was added to saliva. Eight millimolar nitrate is a concentration found in saliva after vegetable intake,^17^ indicating that the positive oral and cardiometabolic effects stimulated by dietary nitrate could be impaired in periodontitis. The effect of periodontal treatment on oral and systemic parameters after nitrate intake should thus be explored in future studies.

Kapil et al.^20^ determined the NRC by letting individuals rinse their mouths with a nitrate-containing solution. Additionally, the NRC could be measured in oral biofilm samples normalized by weight or DNA concentrations. Future studies should compare different methods to determine the NRC, as this could represent a helpful marker of gingival health, and how it correlates with systemic parameters.

In line with the NRC results, we showed that the subgingival nitrate-reducing microbiota decreases in periodontitis (compared with periodontal health) and increases after periodontal treatment. Specifically, we showed this for the relative abundance of known nitrite-producing bacteria, including confirmed nitrate-reducing bacteria. The difference between these two groups is that nitrite-producing isolates are detected by incubating bacteria with nitrate and measuring nitrite production. Most of the time, this nitrite results from nitrate reduction, but there are other pathways that can lead to nitrite production (for example, the oxidation of nitric oxide or ammonium).^44^ The NRC of oral bacteria should thus be confirmed by physiological measurements of nitrate.^7^ In our study, both groups (nitrite-producing and confirmed nitrate-reducing) showed similar statistical significance (i.e., *P* < 0.5 remained *P* < 0.5 when comparing these groups between periodontitis and health or before and after periodontal treatment). Additionally, qPCR measurements of the nitrate-reducing biomarker and periodontal-health-associated genus *Rothia* before and after periodontitis treatment confirmed an increase of *Rothia* cells per sample (*P* < 0.05). In our previous study, it was found that the *Rothia* levels in these samples, which were determined by Illumina sequencing of the 16S rRNA gene, were higher after treatment.^38^ Our data confirms that nitrate-reducing bacteria decrease in the subgingival plaque under the conditions of inflammation and dysbiosis associated with periodontitis. It is also interesting that *Rothia* levels correlated well with the proportion of nitrate-reducing bacteria (Supplementary Datasheet), indicating that this genus is a potential biomarker of nitrate-reducing species. The degree of periodontitis, as indicated by the full mouth bleeding score (FMBS), showed a negative correlation with *Rothia* levels determined by qPCR. This supports a possible role of *Rothia* in periodontal health and its assessment as a potential periodontal probiotic, as recently proposed.^38,48^

### Potential implications of hampered NRC for systemic health

Our data indicate that periodontitis could limit nitrate reduction in the presence of nitrate concentrations found after vegetable intake. The reduction of nitrate by oral bacteria is an essential step in the nitrate-nitrite-nitric oxide pathway that contributes to systemic nitric oxide levels.^20^ When sterilizing a significant proportion of the oral microbiota with chlorhexidine in fasting individuals, the NRC of the oral microbiota was impaired, plasma nitrite levels dropped and blood pressure increased.^20^ Conversely, stimulating the nitrate-nitrite-nitric oxide pathway by consuming vegetable juices or nitrate salts can lead to a decrease in blood pressure and improved endothelial function.^18,52–54^ Nitrate intake has also been associated with a reversal of metabolic syndrome and with antidiabetic effects,^18,55^ while the use of over-the-counter mouthwash was found to correlate with diabetes and pre-diabetes development.^56^ It thus appears that conditions in which a deficit of nitric oxide is found,^55,57^ benefit from stimulating nitrate reduction by the oral microbiota. Remarkably, periodontitis is associated with cardiovascular diseases and diabetes.^58,59^ Future studies should therefore determine if periodontitis can contribute to cardiovascular diseases, diabetes, and other nitric oxide-related systemic conditions by limiting the beneficial effects of dietary nitrate-derived nitric oxide. This possibility is supported by the finding that the genetic capacity of subgingival plaque to produce nitrite is associated with lower levels of cardiometabolic risk.^16^

Pre-eclampsia is another example of a condition associated with both reduced nitric oxide availability^60^ and periodontitis,^10^ while a recent study indicated that this condition was associated with a decrease in oral nitrate-reducing bacteria.^61^ In relation to this, periodontal treatment appears to reduce complications in pregnant women with periodontitis,^62^ possibly by reducing inflammatory and bacterial exposure to unborn babies.^10^ In our study, we found that periodontal treatment recovered the NRC to healthy levels in the presence of 8 mmol/L nitrate. The potential effect of improved dietary nitrate reduction resulting from periodontal treatment on pre-eclampsia should be explored. Periodontal treatment is also associated with a long-term improvement of endothelial function.^63^ In a recent study in mice, it was shown that inorganic nitrate protects against and can partially reverse pre-existing, periodontitis-induced endothelial function through restoration of nitrite and, thus, nitric oxide levels.^54^ These findings show that both nitrate consumption and periodontal treatment can result in improved endothelial function. Nitrate intake should thus be explored as an adjunct treatment to improve systemic parameters in patients with periodontitis.

### Study limitations and future perspectives

Limitations of this study are that individuals were not fasting when donating saliva and plasma samples and did not receive instructions regarding other habits that could interfere with salivary and plasma nitrate and nitrite levels (e.g., exercise habits, and fitness levels, sunlight, menstrual cycle stage, water consumption).^64,65^ Considering the large effect of the diet on plasma and salivary nitrate and nitrite levels, the study design was not ideal to see how periodontitis affects the basal levels of these molecules. This could explain why the basal saliva and plasma levels of nitrate and nitrite were not significantly different before and after periodontal treatment. However, when incubating saliva with 8 mmol/L nitrate, differences were found in the NRC of periodontitis patients before and after treatment and between periodontitis patients before treatment and healthy individuals. In vivo, this would mean that the salivary nitrate concentrations obtained by vegetable consumption are reduced less efficiently in periodontitis, decreasing the beneficial effects of dietary nitrate. In the current study, we did not collect blood samples after conditions of high nitrate availability, which is when the effect of a healthy nitrate-reduction bacterial community is expected to be most relevant. Thus, we propose that future studies should test if exposure to nitrate (e.g., by beetroot juice consumption) has different effects on salivary and plasma nitrate and nitrite levels, as well as their derived systemic effects (e.g., blood pressure reduction) in periodontitis patients (before and after periodontal treatment) and healthy individuals. Finally, in this study, the composition of subgingival plaque was studied because it is most affected by periodontitis. Additionally, it is known that the amounts of a given taxon in different oral samples may correlate with each other (for example, *Porhyromonas gingivalis* in subgingival plaque correlates with *P. gingivalis* in saliva^66^), indicating that changes in subgingival plaque affect other communities to some extent. In future work, the effect of this disease on the composition of other microbial communities involved in nitrate reduction (e.g., the tongue microbiota) should be explored.

### Concluding remarks

In conclusion, our data show that periodontitis compromises the nitrate-reducing microbiota and impairs the reduction of nitrate concentrations that are found in saliva after vegetable intake. However, the impaired nitrate reduction capacity is recovered to healthy levels after periodontal treatment. Given that a diminished or eliminated nitrate reduction capacity, for instance, by antiseptic mouthwash, derives in lower plasma nitrite levels,^20^ periodontal disease could imply a deficit in circulatory nitrite. Future studies should investigate whether, in addition to the oral-systemic link derived from inflammatory effects, a hampered nitrate reduction capacity in periodontal patients could further contribute to the multiple systemic conditions that are affected by nitric oxide availability. Taking into account that nitrates intake increases periodontal health-associated bacteria,^7^ it should be determined if nitrate-rich dietary interventions could improve periodontal treatment outcomes, including oral and systemic parameters.

### Supplementary information

Supplementary Figures and Tables

Supplementary Dataset

## Data Availability

The sequencing reads of the 42 periodontitis patients before (BL) and after (D90) treatment are deposited in the NCBI Sequencing Read Archive (SRA) under BioProject PRJNA725103. Any further data is available upon reasonable request from the corresponding author.
