# Publisher Correction: Nitrate reduction capacity of the oral microbiota is impaired in periodontitis: potential implications for systemic nitric oxide availability

**DOI:** 10.1038/s41368-024-00283-2

**Published:** 2024-01-26

**Authors:** Bob T. Rosier, William Johnston, Miguel Carda-Diéguez, Annabel Simpson, Elena Cabello-Yeves, Krystyna Piela, Robert Reilly, Alejandro Artacho, Chris Easton, Mia Burleigh, Shauna Culshaw, Alex Mira

**Affiliations:** 1 Department of Genomics and Health, FISABIO Foundation, Center for Advanced Research in Public Health, Valencia, Spain; 2 Department of Biological and Biomedical Sciences, Glasgow Caledonian University, Glasgow, UK; 3 Oral Sciences, University of Glasgow Dental School, School of Medicine, Dentistry and Nursing, College of Medical, Veterinary and Life Sciences, University of Glasgow, Glasgow, UK; 4 Sport and Physical Activity Research Institute, University of the West of Scotland, Blantyre, Scotland; 5 Instituto de Biomedicina de Valencia, Consejo Superior de Investigaciones Científicas (IBV-CSIC), Valencia, Spain; 6 CIBER Center for Epidemiology and Public Health, Madrid, Spain

**Keywords:** Dental diseases, Microbiome, Physiology

Correction to: *International journal of Oral Science* 10.1038/s41368-023-00266-9, published online 05 January 2024

Following publication of the original article^1^, the authors reported a typesetting error in figure 1.

In the previously published version, the bullet points under “Treatment” were not correctly structured.

The correct figure 1 should read:
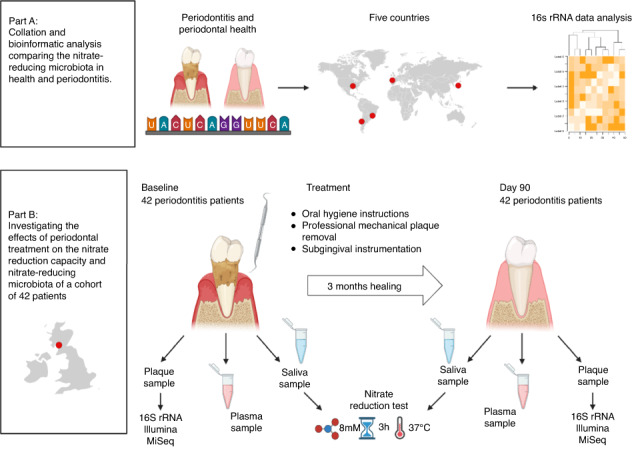


The original article^1^ has been updated.
