# Supplementary material for: Nitrate reduction capacity of the oral microbiota is impaired in periodontitis: potential implications for systemic nitric oxide availability
Source: Int J Oral Sci. 2024 Jan 5;16:1. doi: 10.1038/s41368-023-00266-9 (PMC10767001; doi:10.1038/s41368-023-00266-9)
Supplement: Supplementary file 1 — Supplementary Figures and Tables [file 41368_2023_266_MOESM1_ESM.docx]

**Nitrate reduction capacity of the oral microbiota is impaired in periodontitis: potential implications for systemic nitric oxide availability**

Bob T. Rosier^1*^, William Johnston^2,3*^, Miguel Carda-Diéguez^1^, Annabel Simpson^4^, Elena Cabello-Yeves^1,5^, Krystyna Piela^3^, Robert Reilly^3^, Alejandro Artacho^1^, Chris Easton^4^, Mia Burleigh^4^, Shauna Culshaw^3^, Alex Mira^1,6#^

^1^ Department of Genomics and Health, FISABIO Foundation, Center for Advanced Research in Public Health, Valencia, Spain.
^2^ Department of Biological and Biomedical Sciences, Glasgow Caledonian University, Glasgow, UK.

^3^ Oral Sciences, University of Glasgow Dental School, School of Medicine, Dentistry and Nursing, College of Medical, Veterinary and Life Sciences, University of Glasgow, UK.
^4^ Sport and Physical Activity Research Institute, University of the West of Scotland, Blantyre, Scotland.

^5^ Instituto de Biomedicina de Valencia, Consejo Superior de Investigaciones Científicas (IBV-CSIC), Valencia, Spain.

^6^ CIBER Center for Epidemiology and Public Health, Madrid, Spain.

^*^Shared first author

^#^Correspondaning author: Alex Mira, Department of Health and Genomics, FISABIO Foundation, 46020 Valencia (Spain); tel. +34 961925925, e-mail: mira_ale@gva.es

**Supplementary Information (2 tables, 2 figures)**

*Supplementary Table 1: studies used for bioinformatic analysis to compare the nitrate-reducing microbiota in health and periodontitis.*

| **Study** | **Country** | **16S rRNA gene sequencing platform** | **Healthy individuals** | | | | **Individuals with periodontitis** | | | |
| --- | --- | --- | --- | --- | --- | --- | --- | --- | --- | --- |
|  |  |  | **Criteria used to define health^b^** | **N** | | **PD information** | **Criteria used to define periodontitis^b^** | **N** | | **PD information** |
|  |  |  |  | **Pers.** | **Samples^c^** |  |  | **Pers.** | **Samples^c^** |  |
| *Ikeda et al. (2020)* | Japan | 454 GS-FLX Titanium pyro-sequencing | Having a PD of ≤ 3 mm at all sites. | 10 | 10 | 2.3 ± 0.4 (sampled site) | At least four sites with PD ≥ 6 mm in each quadrant. | 10 | 10 | 6.5 ± 1.2 (sampled site) |
| Pérez-Chaparro et al. (2018) | Brazil | Illumina MiSeq sequencing | A maximum of 10% of the sites with BoP and/or gingival bleeding and no sites with PD and CAL ≥3 mm. | 7 | 21 | 1.9 ± 0.2 (average) | At least 30% of sites with PD and CAL ≥4 mm and a minimum of three teeth with at least one site presenting the following categories: i) PD and CAL ≤3 mm without bleeding on probing (BoP), ii) PD and CAL 4–6 mm with BoP, iii) PD and CAL ≥7 mm with BoP | 9 | 27 (deep pockets) | 3.9 ± 0.7 (average) |
| Abusleme et al. (2013)^a^ | Chile | 454 GS-FLX Titanium pyro-sequencing | At least 90% of sites with PD and CAL ≤3 mm, no site with PD >4 mm and less than 10% of sites presenting BoP | 10 | 17 | 1.5 ± 0.1  (average) | ≥5 teeth with PD ≥5 mm and clinical attachment level (CAL) ≥4 mm, had BoP in at least 20% of sites and showed radiographic evidence of bone loss | 22 | 22 (deep sites) | 3.0 ± 0.7 (average) |
| Griffen et al. (2012) | USA | 454 GS-FLX Titanium pyro-sequencing | No pockets with probing depth >4mm | 29 | 29 | 2.81 ± 0.5 (sampled sites) | At least 4 mm attachment loss and 5 mm probing depth in at least three non-adjacent interproximal sites in at least two quadrants | 29 | 29 (deep sites) | 6.15 ± 1.30 (sampled sites) |
| Camelo-Castillo et al. (2015)^a^ | Spain | 454 GS-FLX Titanium pyro-sequencing | No sites with PD ≥4 mm, no radiographic evidence of alveolar bone loss and BOP <20% | 22 | 22 | 2.35 ± 0.33 (sampled sites) | ≥5 teeth with PD ≥5 mm and CAL ≥4 mm, had BoP in at least 20% of sites and showed radiographic evidence of bone loss | 60 | 60 | 5.85 (sampled sites) |

^a^ Criteria to define periodontitis by Page & Eke (2007)
^b^ Clinical attachment level (CAL), Probing depth (PD) and Bleeding on probing (BoP)
^c^ Samples with FASTQ deposited in NCBI, which were used in our analysis. Some individuals had multiple samples that were all added to healthy or periodontitis group.

*Supplementary Table 2: bacterial species in different groups.*

| **Bacterial group** | **Species^*1^** | **Reference(s)** |
| --- | --- | --- |
| **Nitrite producers^*2^** | *Actinomyces georgiae, Actinomyces graevenitzii, Actinomyces hongkongensis, Actinomyces johnsonii, Actinomyces lingnae, Actinomyces massiliensis, Actinomyces naeslundii, Actinomyces odontolyticus, Actinomyces oris, Actinomyces viscosus, Capnocytophaga gingivalis, Capnocytophaga ochracea, Capnocytophaga sputigena, Corynebacterium durum, Corynebacterium matruchotii, Cutibacterium acnes, Eikenella corrodens, Fusobacterium nucleatum, Granulicatella adiacens, Haemophilus parainfluenzae, Haemophilus segnis, Kingella denitrificans, Neisseria elongate, Neisseria flavescens, Neisseria macacae, Neisseria mucosa, Neisseria oralis, Neisseria sicca, Neisseria subflava, Paraburkholderia fungorum, Prevotella melaninogenica, Propionibacterium acnes, Pseudopropionibacterium propionicum, Rothia aeria, Rothia dentocariosa, Rothia mucilaginosa, Schaalia odontolytica, Selenomonas artemidis, Selenomonas flueggei, Selenomonas noxia, Streptococcus australis, Streptococcus infantis, Streptococcus mitis, Streptococcus mutans, Streptococcus oralis, Streptococcus parasanguinis, Streptococcus salivarius, Streptococcus sanguinis, Veillonella atypica, Veillonella dispar, Veillonella parvula, Veillonella tobetsuensis* | Rosier et al. (2022) |
| **Confirmed nitrate reducers^*3^** | *Actinomyces georgiae, Actinomyces graevenitzii, Actinomyces hongkongensis, Actinomyces johnsonii, Actinomyces lingnae, Actinomyces massiliensis, Actinomyces naeslundii, Actinomyces odontolyticus, Actinomyces oris, Actinomyces viscosus, Cutibacterium acnes, Kingella denitrificans, Neisseria elongate, Neisseria flavescens, Neisseria macacae, Neisseria mucosa, Neisseria oralis, Neisseria sicca,*  *Neisseria subflava, Propionibacterium acnes, Pseudopropionibacterium propionicum, Rothia aeria, Rothia dentocariosa, Rothia mucilaginosa, Schaalia odontolytica, Veillonella atypica, Veillonella dispar, Veillonella parvula, Veillonella tobetsuensis* | Rosier et al. (2022) |
| **Periodontitis associated** | *Acinetobacter baumannii, Aggregatibacter actinomycetemcomitans, Alloprevotella tannerae, Anaeroglobus geminatus, Campylobacter gracilis, Campylobacter rectus, Campylobacter showae, Dialister pneumosintes, Enterococcus faecalis, Escherichia coli, Eubacterium brachy, Eubacterium nodatum, Eubacterium saphenum, Filifactor alocis, Fretibacterium fastidiuosum, Fusobacterium nucleatum, Fusobacterium nucleatum subsp. animalis, Fusobacterium nucleatum subsp. nucleatum, Fusobacterium nucleatum subsp. polymorphum, Fusobacterium nucleatum subsp. vicentii, Fusobacterium periodonticum, Mogibacterium timidum, Parvimonas micra, Peptostreptococcus stomatis, Porphyromonas endodontalis, Porphyromonas gingivalis, Prevotella denticola, Prevotella intermedia, Prevotella nigrescens, Selenomonas sputigena, Tannerella forsythia, Treponema denticola, Treponema lecithinolyticum, Treponema medium, Treponema vincentii* | Socransky et al. (1998); Pérez-Chaparro et al. (2014) |
| **Red complex^*4^** | *Porphyromonas gingivalis*, *Tannerella forshytia*, *Treponema denticola* | Socransky et al. (1998) |

^*1^In each dataset, the species in these lists that were found were added to these groups
^*2^ Nitrite-producing species were found to produce nitrite in the present of nitrate, but it was not always confirmed if the nitrite production was due to nitrate reduction
^*3^ Confirmed nitrate reducers were considered all nitrite-producing species from genera with confirmed nitrate-reducing representatives
**^*4^** Previously reported and analysed by Johnston et al. (2021)

**
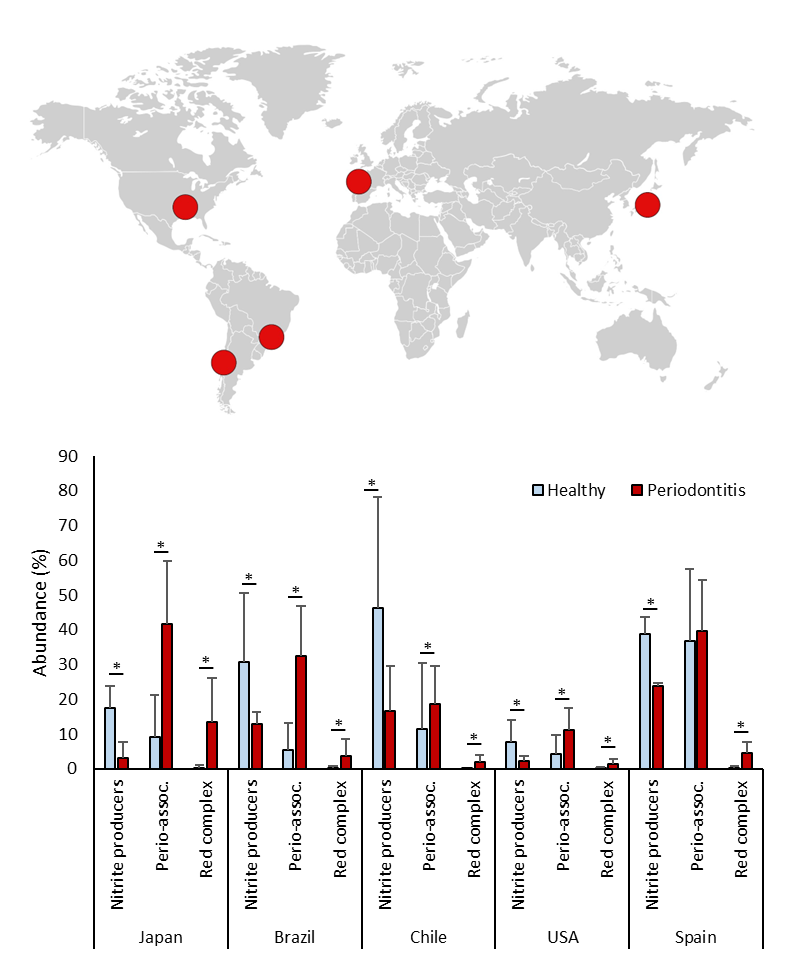
**

**Supplementary Figure 1: Nitrite-producing bacteria in periodontitis and health.** Bargraphs show the relative abundance of bacteria in subgingival plaque samples from different countries, as estimated by high-throughput sequencing of the 16S rRNA gene. Bacteria were grouped in nitrite-producing species, periodontitis-associated or “red-complex” periodontal pathogens according to Rosier et al. (2022), Pérez-Chaparro et al. (2014) and Socransky et al. (1998), respectively (the bacterial species in each group are listed in Supplementary Table 2). Healthy individuals (blue bars) were compared with individuals with periodontitis (red bars). The datasets include individuals from Japan, Spain, USA, Brazil and Chile (n = 20-82 per dataset, see Supplementary Table 1 for additional information). It should be noted that the levels of groups of bacteria in different studies are affected by the criteria used to describe periodontitis, regional and host factors, as well as the DNA extraction methods or the sequencing techniques used in the original studies. *adjusted p < 0.05 of compositional data standardized by ANCOM-BC and compared with a Wilcoxon test.


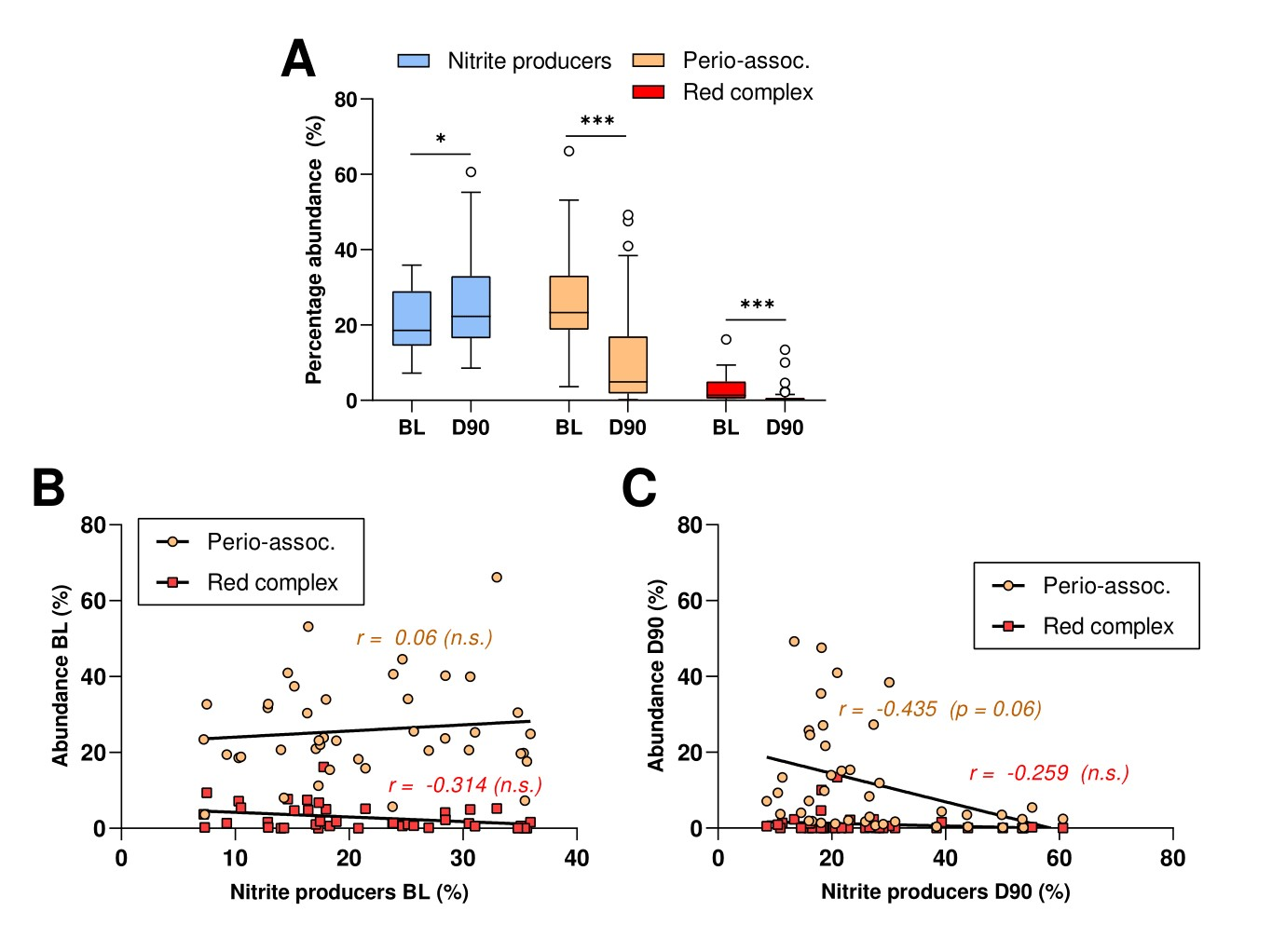


**Supplementary Figure 2: Nitrite-producing species and disease-associated bacteria before and 90 days after periodontal treatment.** A) Relative abundances of nitrite-producing bacteria, red complex and periodontitis-associated bacteria before (baseline, BL) and 90 days after treatment (D90) of 42 periodontitis patients. *adjusted p < 0.05, *** adjusted p < 0.001 of compositional data standardized by ANCOM-BC and compared with a Wilcoxon test. B and C) Correlations between abundance of periodontal pathogens and nitrite-producing bacteria at baseline (BL) and 90 days after treatment (D90). *adjusted p-values of Spearman's rank correlation reported (n.s. = not significant).
